# COVID-19: its consequences and lessons

**DOI:** 10.47626/1679-4435-2020-184

**Published:** 2021-03-03

**Authors:** Mírian Perpétua Palha Dias Parente

**Affiliations:** Editor-in-Chief, *Revista Brasileira de Medicina do Trabalho*

The unexamined life is not worth living.

Socrates

“Time flies...” and thus we came to the end of 2020, a most unusual year that has changed the course of history. The pandemic of coronavirus disease 2019 (COVID-19), caused by the SARS-CoV-2 virus, has left us shaken and deeply scarred all of our lives, as well as warned us of the urgent need for cooperation between nations.

Since it was first reported in December 2019, the severe acute respiratory syndrome caused by the novel coronavirus (SARS-CoV-2) has spread globally, infecting almost 73 million people and leading to more than 1,600,000 deaths worldwide as of the time of writing.

The first recorded case of COVID-19 in Brazil was reported on February 25, 2020, and for the rest of the year, with the exception of the electoral period, the COVID-19 pandemic declared by the World Health Organization^1^ was the chief issue on everyone’s minds.

We have begun to live with the new normal, that is, within a proposed new standard that might ensure our survival. Highly technical issues such as the urgent need for scientific evidence to support the use of medicines and vaccines, mask mandates, and hand sanitizers have become the object of popular discussion; social distancing and social isolation, increasingly widespread use of the term *lockdown*, school and university closures, bans on religious worship and social events, an increasing portfolio of public health interventions, and so many other measures meant to protect us by preventing mass gatherings have also caused us to lose touch with our loved ones. Coworkers, friends, and relatives have been killed by a fearsome virus that spread worldwide and challenged the most renowned infectious diseases specialists, the most eminent epidemiologists, and all those who believed they had mastered science.

In his *Toilers of the Sea* (1866), French playwright and novelist Victor Hugo (1802-1885) averred that “Sublime characters are stubborn. A man who is merely brave has only one method of action, a man who is merely valiant has only one temperament, a man who is merely courageous has only one virtue; greatness is reserved for the man who is stubborn in pursuing the right course”.^2^ This, perhaps, is how we might classify the vast majority of health care workers during the COVID-19 pandemic. They stubbornly looked for ways to better care for their patients, in the midst of total ignorance regarding how the disease would manifest itself, until a measure of tranquility was obtained with advancing knowledge on respirator management, prone positioning, and medication strategies to provide appropriate support according to the needs of each patient.

In the field of occupational health, major issues came to the fore, especially with the legalization of telemedicine for the duration of the pandemic and the ethical issues raised by *tele-examination* of workers and the consequent transformation of the doctor–patient relationship. Ethical and legal principles must be safeguarded for the physician, the patient (worker), and the employer. Another very difficult decision involved defining who would *telecommute*, or work from home, and when to place individuals in high-risk groups on leave from their face-to-face activities; the logistics of contact tracing and isolation were also challenging.

The greatest concerns in relation to health care workers certainly involved adequate protection so that they could discharge their functions safely. Front-line workers are more exposed to the virus and to excess workloads, largely due to a lack of personnel. This surge of patients was further compounded by supply shortages and difficulties in testing. Most professionals have had to endure isolation from their families and learn to cope with the loss of those who have died, affecting them emotionally and physically.

Although COVID-19 represents an enormous burden on the economy, the health system, and society, 2020 came to an end with excellent news: the start of vaccination in other countries and the prospect that an effective and safe vaccine will soon be available to all. Many challenges remain, especially the possibility of emerging variants of the epidemic strain of SARS-CoV-2 giving rise to new mechanisms associated with increased spread across human populations.^3^ However, from a positive standpoint, the fact that we are alive and have survived the pandemic means we can say goodbye to 2020 with gratitude, seek out more wisdom and the ability to enjoy a new outlook on life, and welcome 2021 with the hope of a better future.

Happy 2021!
